# At first glance, informal payments experience on track: why accept or refuse? Patients’ perceive in cardiac surgery department of public hospitals, northeast of Iran 2013

**DOI:** 10.1186/s12913-017-2108-4

**Published:** 2017-03-14

**Authors:** Ali Vafaei Najar, Hossein Ebrahimipour, Arefeh Pourtaleb, Habibollah Esmaily, Mehdi Jafari, Zohre Nejatzadegan, Yasamin Molavi Taleghani

**Affiliations:** 1 Department of Management Sciences and Health Economics, School of Health, Social Determinants of Health Research Center, Mashhad University of Medical Sciences, Mashhad, Iran; 2 Health Management and Economics Research Center, Iran University of Medical Sciences, Tehran, Iran; 3 School of Health Management and Information Sciences, Iran University of Medical Sciences, Tehran, Iran; 4 Biostatics and Epidemiology Department, School of Health, Social Determinants of Health Research Center, Mashhad University of Medical Sciences, Mashhad, Iran; 5 Health Management and Economics Research Center, Isfahan University of Medical Sciences, Isfahan, Iran

**Keywords:** Informal payments, Hospital, Mashhad, Iran

## Abstract

**Background:**

Patient’s Informal payments is among the main source of health care financing in some countries. This paper aimed at determining the patient informal payments and relative factors in Cardiac Surgery Departments (CSD) in hospitals affiliated to Mashhad University of Medical Sciences (MUMS) in 2013.

**Methods:**

In this cross-sectional study, 316 discharged patients were selected using multi-stage sampling. Data gathering tool was a questionnaire which was filled by structured telephone interviews. We used quantitative content analysis for open-ended questions besides descriptive statistics and nonparametric tests by SPSS 16 at 0.05 Sig level.

**Results:**

Sixteen (5.93%) patients made voluntary informal payments. The purpose of payment was: “gratitude” (43.75%), satisfaction with health services provided” (31.25%) and (18.75%) for better quality of services. About 75% of the payments were occurred during receiving health care services. The main causes were “no request for informal payments” (98.14%), “not affording to pay for informal payments” (73.33%) and “paying the hospital expenses by taking out a loan” (55.91%). Responders said they would pay informally in demand situation (51.85%) just for patient’s health priority, 40.71% would also “search for other alternative solutions” and 27.33% “accepted the demand as a kind of gratitude culture”. Twenty four patients (8.9%) had experienced mandatory informal payments during the last 6 months. The minimum amount of payment was 62.5$ and the maximum was 3125$. There was a significant relationship between the way of referring to medical centers and informal patient's payment (*P* ≤0.05).

**Conclusion:**

Despite the widespread prevalent belief about informal payments in public hospitals —particularly to the well-known physicians — such judgment cannot be generalized. The main reasons for the low informal payments in the current study were the personality characteristics of the physicians and hospital staff, their moral conscience and commitment to professional ethics, cultural factors and social-economic status of the patients. Health care system should notify people about their rights specially the payments calculation mechanism and methods. Better communication with the public and especially the media can help to correct attitude toward these payments.

## Background

Cardiovascular diseases, Cancer, Diabetes and Chronic Respiratory Diseases are responsible for majority of deaths in patients suffering from non-communicable diseases (NCDs). Although a focus on strengthening protective factors and early prevention of these diseases particularly cardiovascular diseases is very effective, in many countries such as Iran most financial resources have been devoted to treatment options [1, 2].

There are three main health financial resources in Iran including public revenue, social health insurances and individual’s out of pocket payments [3, 4]. Informal payment is a part of out of pocket payments and it is a distinctive feature of health systems in many countries around the world [5]. Such a payment is a source of inequity and inefficiency in a health system [6]. The definition of informal payments varies across the literature, which reflects the socio-cultural connotations of the term in perceiving it [7, 8].

This phenomenon is both diverse and widespread and has many forms, ranging from cash payments to non- monetory offers and from giving gifts to informal charging. However, consensuses among researchers on the definition of informal payments have been impeded national survey to describe a distinctive-feature and all forms of this phenomenon [7].

There is an increasing interest in the issue of informal payments for health care in transition economics, low and middle –income countries [9–12], to which Iran is not an exception [13, 14]. These payments can pose risks to equity, efficiency and quality of health care provided [15].

An informal payment is a multiple-cause phenomenon with various possible causes such as insufficient source of healthcare funding; deficiency in public budgeting; lack of transparency in health system, accountability, and auditing; low salaries paid to health service providers; poor management; low quality of services; poor access to medical care, social processes and value system; cultural tradition; violation of medical ethics; etc. [7, 9, 12, 13, 16, 17]. A recent approach to informal payment in Iran suggested setting real tariffs, minimizing the direct physician-patient financial relationship, supporting the insurance companies and increasing physicians’ salaries [14]. In Urimia hospitals (Urmia is the second largest city in the Iranian Azerbaijan and the capital of West Azerbaijan Province) the mean informal payment to the doctors was about 157$. Moreover, the mean informal payments to the nurses and other staff were 6.25$ and 5$, respectively [18].

Another study showed that the crucial causes of informal payments are cultural issues, looking for accessing better services with higher quality and legal problems [13].

In Tehran, 21% of the patients reported making informal payments to the hospital staff and 3.7% of the patients reported being asked to make such payments [19].

Another study conducted in Iran during April 2014 showed that about 48% of the respondents reported at least one experience of making informal payment for healthcare in the last year to the study [20].

Study on public attitudes towards informal payments conducted in six central and Eastern European countries (Bulgaria, Georgia, Lithuania, the Netherlands, Romania and Ukraine) showed that there was a negative attitude towards these payments. A majority regarded informal cash payment as corruption but in-kind gifts are considered as token of gratitude. However, significant differences among countries are observed [21]. Another study showed that informal payments in Azerbaijan comprised 84% of the total health system costs [12].

Despite the hidden nature of informal payments and its prevalence, its real scale and scope are yet unknown and its estimations vary in different studies [22]. However, it is a highly controversial challenge to gather valid data in this area for the sensitive nature of the phenomenon and to reflect the dark side of the health care system in each country [23].

Given the insufficient evidence in Iran, particularly as there have not yet been other studies in Mashhad, in this study we addressed the issue of informal payments defined as unofficial cash or in kind payments voluntary (such as gifts or tips) or mandatory (such as bribery) given to the health care personnel in the cardiac surgery departments in hospitals affiliated to MUMS (Mashhad University of Medical Sciences) in 2013. Mashhad is the second most populous city in Iran and the capital of Razavi Khorasan Province. It is located in the northeast of Iran with a population of about 3 million people and accommodates around 20 million pilgrims a year. It is also the medical hub of the north east Iran, where the patients go to the specialty and subspecialty hospitals in this city, which are considered among the most visited national health units in the country [24] Therefore, the study is an attempt to Patients’ informal payments, their experience and also their attitudes towards these payments.

## Methods

### Setting and samples

This descriptive, analytical and cross-sectional study was carried out in April to September 2013. The statistical population consisted of the patients who have undergone a surgery in the Cardiac Surgery Departments (CSD) of big teaching-tertiary hospitals affiliated to MUMS. A multi-stage random sampling (2 stages) was used. In the first stage, 3 hospitals with cardiac surgery units were identified and then the sample size for each hospital was determined based stratified sampling

The previous studies show that about 25% of the people admitted to hospitals made informal payments [19]. Based on the above findings and using the following formula, a sample size of 288 patients was investigated with the confidence level of 95% and an error of less than 5%. Due to possibility of loss, the size of the sample was then increased to 316 patients.

$$ \mathrm{n}={{\mathrm{z}}^2}_{1-\alpha /2}*\mathrm{P}*\left(1-\mathrm{p}\right)/{\mathrm{d}}^2 $$

From a total of 316 patients, 237 patients were from hospital A and 79 patients were from hospital B. Unfortunately, the third hospital refused our official request to participate in this research.

### Data collection

All data were collected by structured telephone interview using a standard questionnaire [19]. Informal payments were explained by the interviewers to the respondents (the patients or their representative) and were asked if they made any voluntary or mandatory payments (in cash or in other forms) to the healthcare providers for any reason in addition to the official costs. The questionnaire consists of four sections. The first section consisted of eight questions about the demographic and health insurance status of the patients, the way of going to the hospital, the length of stay, experience of informal payment and payment time (whether it was before or after the admission).

The second section (answered by those who had made an informal payment to the physicians in the clinic before the admission) comprised of five questions about the types of payment (five forms including offer of flowers and gifts, cash, goods, commitment to work, other), the form of payment (voluntary or compulsory), the approximate amount of money, frequency, and the payment purpose (open question).

The third section (answered by those who had made an informal payment in hospital) included the frequency of payment, the characteristics of the informal payment and the purpose of payment (open questions).

The fourth section was answered by those who had not made any informal payments. These questions determined if there was any request by physicians and/or other hospital staff, the reasons why patients did not pay if they had been asked to do so and then the reasons to their unwillingness to make voluntary payments for those respondents who did not face any request. The other question was about what they would do if they had been asked to pay informally. Finally, the last questions were about patient experience to have mandatory informal payments during the last 6 months. All payments were converted to US dollars based on the average exchange rate during the research period.

We used telephone-survey which is a suitable to cheaply and quickly collect data from geographically scattered samples. Also, this could help to have better communication with patients regardless of the cultural barriers. The minimum length of each phone call was 10 min.

### Data analysis

The data were analyzed using descriptive statistics (frequency, percentage, mean and standard deviation), Mann-Whitney and Kruskal-Wallis tests by SPSS 16 at 0.05 Sig level. The respondents’ answers concerning the reasons for their purpose (qualitative data) were analyzed using quantitative content analysis [25]. The resulting rules are detailed in a coding form by a team of researchers which specifies how and what to code. In preparing the code hierarchy, we made each category exhaustive and mutually exclusive and that the instructions for the coding were clear. The coding was carried out by two coders (ZN and YMT). The reliability was tested by getting coders to code the same set of questionnaires. Descriptive statistics were used for the final analysis of the frequency of codes.

## Results

Out of the 316 phone calls, 270 patients answered the questionnaire. As we used data in both patient's medical records and interview, the total number varies in education, length of stay and way of referring lines. The distribution of the patients with respect to the study variables is shown in Table 1.

**Table 1:** Descriptive statistics of the patients

Demographic characteristics		Number (%)	Total
Gender	Male	172 (54.4)	316
	Female	144 (45.6)	
Age	0–5	36 (11.4)	316
	6–15	22 (7.0)	
	16–29	15 (4.7)	
	30–39	14 (4.4)	
	40–49	26 (8.2)	
	50–59	76 (24.1)	
	More than 60 years	127 (40.2)	
Education	Not in school age	39 (12.3)	286
	Illiterate	132 (41.8)	
	Primary school	69 (21.8)	
	Secondary school	21 (6.6)	
	High school	17 (5.4)	
	Higher	8 (2.5)	
Length of stay (days)	1–7	119 (44.7)	270
	8–14	97 (36.1)	
	15–21	35 (12.9)	
	more than 21	19 (7.0)	
Social status	Infants or children	39 (12.3)	316
	Single	35 (11.1)	
	Married	214 (67.7)	
	Other (widow-divorced)	28 (8.8)	
Employment status	Infants or children	39 (12.3)	316
	Student	27 (8.5)	
	Employee (salaried)	28 (8.9)	
	self-employment	58 (18.4)	
	Laborer	14 (4.4)	
	Farmer	30 (9.5)	
	Housekeeper	106 (33.5)	
	Unemployed	14 (4.4)	
Residency	Mashhad	128 (40.5)	316
	Village	95 (30.1)	
	Towns	65 (20.6)	
	Capital of province	23 (7.3)	
	Foreign	5 (1.5)	
Health insurance status	Insured	292 (92.4)	316
	Uninsured	24 (7.6)	
	have complementary health insurance	59 (18.4)	316
	Without complementary health insurance	257 (81.3)	
Way of referring	Going directly to the clinic	90 (33.3)	270
	Physician’s office	76 (28.1)	
	Referrals from other health centers	54 (20.0)	
	Emergency	50 (18.6)	

Based on the patients’ medical records and their answers, the majority of patients (54.4%) were male and older than 60 years (26.6%). The mean ages for men and women were 49.95 ± 2.45 and 47.06 ± 2.51, respectively. 67.7% of the patients were married, 33.5% housewives and 4/4% unemployed. 92.4% patients were covered by insurance plans. Also, 84.4% of the respondents were rellies and 15.6% were patients.

The purpose for informal payments is presented in Table 2. It is apparent that expressing “gratitude” in form of offering sweets was the first and the second cause was expressing “satisfaction with behavior, performance and handling of patients,” in form of cash payment with the average of 8.12$. “Better services quality” was the other reason for patients' payments. 75% of the payments were made during the health care services providing and 25% were after receiving health care services.

**Table 2:** Distribution of patients’ purposes for informal payments

Purpose for informal payment	Type of informal payment		N	%	Total	
					N	%
Expression-of- Gratitude	Sweets	Physicians	3	42.85	7	43.75
		Nurses	4	57.14		
satisfaction with health care services provided	Cash with an average of 8.12 $	Nurses	1	20	5	31.25
		Guards	1	20		
		Servants	3	60		
Getting better service quality	Goods	Operating room personnel	1	33.33	3	18.75
	Cash with an average of 12.5$	Servants	1	33.33		
		Nurses	1	33.33		
Satisfaction with hospital	Cash (6.25$) to the hospital				1	6.25
Total					16	100

Table 3 points out to the respondents’ reasons who made no informal payments and their frequency. It declares that three main reasons for not having informal payments were “there was no request for informal payments” about 98%, “cannot afford to pay for informal payments” 73.3% and “taking out a loan to pay the hospital expenses” approximately 56%.

**Table 3:** Frequency distribution of respondents’ reasons who made no informal payments

Statement coding	Number	Percent
There was no request for informal payments.	265	98.14
I could not afford to pay for informal health care payments.	198	73.33
I paid the hospital expenses by taking out a loan.	151	55.91
Personnel getting their salaries in return.	111	41.12
I did not accept to make informal payments due to religious beliefs.	94	34.81
There was no need for extra services.	93	34.44
Informal payments suit rich people in private hospitals.	67	24.81
I was obliged to pay for official patient payments not informal payments.	64	23.70
It is unfair to make informal payments.	57	21.11
I am not willing to pay more money as informal payments.	55	20.37
Informal payments are not common in these hospitals.	53	19.62
Informal cash payments to health care personnel are the same as corruption.	49	18.14
It is not accepted with regard to the physician’s dignity.	28	10.37
I offered in-kind thank you gifts to the physicians and staff but they did not accept them.	25	9.25
I would offer something as gratitude if I could afford it.	20	7.40
I sold my house and other assets to pay the formal costs.	18	6.66
I would pay it if I had been asked for it.	15	5.55
There was no need to pay informal as I had some acquaintance working in that hospital.	12	4.44
The patient died and no more health care services was be needed.	10	3.70
Others	6	2.22

The respondent’s answers to the question “what would you do if you were asked to pay informally?” were positive by 140 people (51.85%). It means that the majority of the patients would make informal payments if they were in an urgent situation to pay for essential healthcare services. People also said that they would “search for other alternative solutions” 40.71% and 27.33% “accepted the demand as a kind of gratitude culture”. Motivation for paying informally in demand situation has been shown in Table 4.

**Table 4:** participants’ answer to make informal payment in demand situation

If there was a demand to have informal payment	N (%)	Motivation for paying informally in demand situation	N (%)
Refuse	130 (48.15)	--------------------------------------	--------
Accept	140 (51.85)	Patient’s health priority	140 (100)
		Searching for other alternative solutions	57 (41)
		Gratitude culture	38 (27.33)
		Famous physician, better health care services	35 (25.17)
		Reporting this demand to legal authorities	22 (15.82)
		Physician’s right to ask for more money in treatment process	6 (4.31)
Total	270 (100)		

It is apparent from Table 5 that there is no significant relationship between informal payments and demographic characteristics such as age, gender, residency, insurance coverage, duration of stay and education, but there is a significant association between the way of going to medical centers and making informal payments (*P* ≤ 0.05). Mann-Whitney test was used for binary variables and Kruskal-Wallis for others (*P* ≤ 0.05).

**Table 5:** The statistical relationship between informal payments and demographic characteristics of the patient

Research variable		Mean ± SD	*P*-value
Gender	Male	0.09 ± 0.86	0.13
	Female	0.42 ± 2.00	
Age	0–5	0	0.61
	6–15	0.58 ± 2.34	
	16–29	0.31 ± 1.21	
	30–39	0	
	40–49	0	
	50–59	0.41 ± 1.89	
	More than 60	0.20 ± 1.51	
Length of stay	1–7	0.23 ± 1.44	0.12
	8–14	0.10 ± 0.97	
	15–21	0.28 ± 1.63	
	More than 21 days	0.95 ± 3.08	
Marital status	Infant or child	0	0.49
	Single	0.46 ± 1.88	
	Married	0.28 ± 1.65	
	Widow	0	
Education	Not in school age	0	0.36
	Illiterate	0.33 ± 1.82	
	Primary school	0.15 ± 1.19	
	Secondary school	0	
	High school	0.48 ± 1.95	
	Higher	0.66 ± 1.77	
Residency	Mashhad	0.36 ± 1.86	0.73
	Capital of province	0.44 ± 2.04	
	Town	0.14 ± 1.07	
	Village	0.11 ± 1.01	
	Foreign	0	
Employment status	Infant or child	0.26 ± 1.58	0.89
	Student	0.21 ± 0.99	
	Employee	0.39 ± 1.74	
	Self-employed	0	
	Laborer	0	
	Farmer	0.34 ± 1.80	
	Housekeeper	0.36 ± 1.94	
	Unemployed	0	
Health Insurance coverage	No	0.66 ± 2.50	0.28
	Yes	0.21 ± 1.41	
Type of health insurance	Rural health care	0.20 ± 1.38	0.39
	Social Security	0.13 ± 1.12	
	Iranian health care	6.696 ± 0.956	
	Employee health services	35.730 ± 12.695	
	Armed Forces	0	
	Health care of other sectors	0	
	Aid Committee	0	
	Private insurance	0	
	International Recommendation Letter	0	
	Without Health Insurance	26.001 ± 7.211	
Way of referring	Going directly to the hospital's clinic	0.21 ± 1.41	0.04
	Physician’s office	0.12 ± 1.08	
	Referrals from other health centers	0	
	Emergency	0.73 ± 2.56	
Complementary health insurance	No	0.23 ± 1.40	0.81
	Yes	0.26 ± 1.82	

Eventually, we found that 24 patients (8.9%) had made informal payments in cash during the last 6 months to the study in other public and private hospitals. According to what 19 patients reported (5 patients refused to say the amount of money they had paid), the majority of patients’ payments (36.8%) was 125–156.2$, 21.5% paid between 187.5 and 218.7$ and the third group (15.7%) had paid 2187.5–3125$ as mandatory informal payments. The minimum payment was 62.5$ and the maximum 3125$. All these payments been made before the health care services were provided to the patient.

## Discussion

This study was a descriptive, analytical and cross-sectional research which aimed at determining the conditions of informal payments made by patients and factors exerting influence on it in cardiac surgery departments of hospitals affiliated to the Mashhad University of Medical Sciences in 2013.

According to our results, about 6% of the sample made voluntary and 94% made no form of informal payments at the hospitals or physician's offices with the main reason “no request or demand” for an informal payment. This result is in somehow compatible with Imani et al. study which aimed to analyze the costs of cardiovascular diseases in another educational public hospital in Tabriz (capital of East Azerbaijan Province, Iran) 2015. They found that there were no instances of an informal payment to clinical or administrative personnel and health service providers in hospitalized patients [26]. Other studies showed that in Tajikistan 20–40% and in Bulgaria 33% of the clients reported to have informally paid for hospitalizations in surgical departments [27, 28]. Nearly 35%–60% in each European country and 15% of people in Russian had made informal payments [21, 29].

Informal payments for receiving maternity health care services in public hospitals in Greece showed a high rate of this sort of payment (74.4%) [30]. The results of another study showed that informal cash payments are common for surgeries, childbirth and skipping waiting lines for diagnostic tests [31].

Although no published research was found on the scale and scope of these payments in Iran, unofficial reports estimate that 5% of the physicians receive informal payments. In two other studies concerning Iran, conducted in Tabriz and Tehran, 10% and 21% of the patients reported to have made informal payments, respectively [19, 32]. A recent survey of training hospitals in Shiraz showed that 20% of the patients experienced informal payments [33].

This difference could be due to selection of the patients from various departments such as General, Surgical, Internal, Emergency and ICU and CCU, but here we focused on informal payments just in the cardiac surgery departments. In this regard, it should be noted that the frequency of informal payments can be different according to the type and nature of the health care services. Paying informally is more common in emergency, life threatening and vital medical procedures like critical care units and operation units [19, 30].

In this regard, it seems that in our study the main reasons for the low payments are the culture, personality traits of the physicians and staff working at these hospitals, work ethic and professional and moral commitments, as emphasized by participants. Nekoeimoghadam and colleagues also showed that social and cultural reasons were the most important factor in experiencing rather rare cases of informal payments in Iran. This factor has been mentioned in other international studies, too [13, 21, 34, 35].

According to the patients’ statements, it seems that another cause is the socio-economic status of patients who are admitted in these hospitals. A majority of the patients are among the vulnerable, low-income and rural people and the main reason for visiting the hospitals is the very limited financial ability to pay for medical expenses let alone pay any informal payment. In other word, they are rural people and others of low socio-economic status who cannot afford to pay the high treatment expenses and/or make informal payments for receiving health care services. Meskarpour et al. found that the patients’ socio-economic status can significantly affect the likelihood and frequency of informal payments for health care services [20]. In Nigeria, informal payments were observed among all people with different socio-economic characteristics in both rural and urban areas [34].

Findings show that there was no significant relationship between the patients’ informal payments and their demographic characteristics such as age, gender, residency and education, which is in line with the studies carried out on Shiraz and Tehran [19, 32]. This finding in our population also is consistent with the Turkish and Lithuanian people [5, 17]. In contrast to our study, Meskarpour et al. found that older people, members of small and wealthier families, employed people and those who are under coverage of only basic medical insurance are more at risk of making such payments [20]. So the policymakers should follow scientific results with more consideration given to socio-economic and demographic factors in order to improve the effectiveness of the policies in this field. It means that informal payments could be perceived and made based on cultural, economic and social situations in any regions.

In this study researchers found that there was no relationship between patients’ health insurance and informal payments, which was consistent with the study on Tehran [19]; however, in Shiraz hospitals, informal payments were found to be significantly more prevalent among the primarily insured patients (*P* < 0.05) [32]. In Iran, more than 90% of the people are covered by health insurance, but informal payment exists in many health sections [31]. Hacer Özgen and colleagues showed that health insurance does not seem to provide financial protection against informal payments [5].

Generally, in present study, cash offering was the most frequent form of payment consist with other studies [19, 32, 33]. Findings in Turkey showed that 69.7% of informal payments were made in cash, 29.4% in non-cash and 0.89% in gifts such as flowers, chocolate, etc. [5].

The findings showed that the payment form of giving sweets to the medical personnel with the purpose of “gratitude” had the highest frequency, which is consistent with the studies on Shiraz and Tehran [19, 32]. The results of the hospitalized cardiac patients in Tabriz 2010 showed that 20% of the informal payments was made as gratitude and 80% upon the physician s’ requests [31]. Other international researches in countries such as Bulgaria, Albania, Ukraine, Slovenia and the Czech Republic confirm informal payments with this purpose [6, 23, 34, 36]. The “gratitude” has also been defined as a motivation for informal payments [37].

Similarly, evidence shows that in expressing gratitude, gifts are more commonly given after the treatment and money often before or during the treatment [37], but here, the purpose for gratitude was all made in form of offering sweets and flowers and not in cash. Patients pay money or offer gifts to show their gratitude. The clients believe that when someone does good things for them, they have to do their best for him/her in return.

Findings show that in-cash informal payments were mostly paid to servants to show “satisfaction with healthcare services provided”. We can conclude that due the shortage of welfare facilities in public educational hospitals and also servants’ low salaries, the informal payments can be made in the hope of receiving better services or appreciating the services provided by the servants and also their behavior and performance. The majority of the patients and their families have a closer relationship with the ‘services section’ personnel than other hospital staff and this increases the chance to undergo voluntary payments (especially for gratitude). On the other hand, the expectations and needs of the services section personnel are lower and more homogeneous with the patients’ financial status.

Consistent with Tehran hospitals study [19], this study revealed that the last type of informal payment was made to receive higher service quality in form of goods. None of the respondents reported the work commitment. In Greece, 42% of those who reported making informal payments stated that it was because of the fear of receiving substandard care with low quality. Informal payments have been reported in other countries such as Albania, New Zealand, Greece and Tajikistan to better access and enhance the quality of or provide additional health care services [17, 30, 34, 36, 38].

The present study showed there were no informal payments (including payments in cash) to the physicians at the hospitals studies. According to the findings, the way of referring to the hospital’s clinic had the highest frequency while there was a significant relationship between variables the way of referring to hospitals and informal payment. Although these findings can be considered as a noticeable achievement, undoubtedly they reveal that more comprehensive studies would be needed in this field. Most physicians at these public training hospitals were the faculty members who did not have long-term presence at the hospital. Furthermore, most treatments are performed by residents. This reduces the frequency of direct communication between the physician and the patient, which can partially explain the low frequency of payments to the physicians. Despite enjoying fame and expertise in performing cardiac surgery and despite the willingness on the part of the patients to make informal payments, the physicians in this study did not receive any offers, seeing it violating their morals and ethics. This is in line with other studies [26, 31, 39]. This can reaffirm that still medical treatment is a humanitarian and religious duty and through a deeper look it could be deemed as a kind of saying prayers and getting closer to God and serving the other people [36, 37]. Rightly, it could be held that many of the physicians carry out their responsibilities quite properly.

### Limitation of the study

The hidden dimensions of informal payments make it difficult to offer an accurate measurement and in-depth analysis of the issue. Not only there are not official reports and documents pertaining to the phenomenon, but also it is done illegally, so both sides of the payment, the patients and the receivers, are unwilling to talk about it. On the other hand, the particular relationship between the patient and physician or health care provider on the one hand, and the continuous process of treatment for some respondents on the other, may have hindered the patients to express the reality in some cases due to fear of negative consequences on services they receive in future. Although the researchers were serious about determining the statue of informal patient payments in cardiac surgery departments in question, we should generalize results of this study to other hospitals in the country as a whole cautiously, considering the fact that the hospitals here are not a real sample of the Iranian society.

## Conclusion

Before attempting to address the issue of informal patient payments, it is necessary to understand the causes. Despite the widespread prevalent belief about informal payments in public hospitals, particularly to the well-known physicians, such a judgment is not generalizable the socio-cultural factors and the governing organizational atmosphere has a significant impact in occurring the informal payments in hospitals. The main reasons for finding low payments in the present study are the personality traits of the physicians and staffs, work ethics and their professional and moral commitments. The other cause can be socio-economic status of the patients admitted to these public educational hospitals in Iran. The majority of these patients are among the low income groups or coming from the rural area that purposefully opt for these hospitals as they already know or have heard there would be no informal payments. Policy makers should pay attention that different factors such as moral, religious, legal and especially cultural issues are behind any informal payment in Iran. Trying to change the established opinions that deem informal payments necessary besides systematic organizing of voluntary gifts, aids or subsidies of patients can be an effective way to control and decreases informal payments. It would be essential to put up social resistance against this type of payment, so health care system should notify people about their rights specially the payments calculation mechanism and methods. Better communication with the public and especially the media can help to correct this attitude toward these payments.

## Abbreviations

CSDs: Cardiac Surgery Departments
MUMS: Mashhad University of Medical Sciences
NCDs: Non-Communicable Diseases
